# Using water–solvent systems to estimate in vivo blood–tissue partition coefficients

**DOI:** 10.1186/s13065-015-0134-z

**Published:** 2015-10-16

**Authors:** Caitlin E. Derricott, Emily A. Knight, William E. Acree, Andrew SID Lang

**Affiliations:** Computing and Mathematics Department, Oral Roberts University, Tulsa, OK 74171 USA; Department of Chemistry, University of North Texas, 1155 Union Cir, Denton, TX 76203 USA

**Keywords:** Blood–tissue partition coefficients, Abraham model, Blood–brain barrier, Pharmacokinetics

## Abstract

**Background:**

Blood–tissue partition coefficients indicate how a chemical will distribute throughout the body and are an important part of any pharmacokinetic study. They can be used to assess potential toxicological effects from exposure to chemicals and the efficacy of potential novel drugs designed to target certain organs or the central nervous system. In vivo measurement of blood–tissue partition coefficients is often complicated, time-consuming, and relatively expensive, so developing in vitro systems that approximate in vivo ones is desirable. We have determined such systems for tissues such as brain, muscle, liver, lung, kidney, heart, skin, and fat.

**Results:**

Several good (p < 0.05) blood–tissue partition coefficient models were developed using a single water–solvent system. These include blood–brain, blood–lung, blood–heart, blood–fat, blood–skin, water–skin, and skin permeation. Many of these partition coefficients have multiple water–solvent systems that can be used as models. Several solvents—methylcyclohexane, 1,9-decadiene, and 2,2,2-trifluoroethanol—were common to multiple models and thus a single measurement can be used to estimate multiple blood–tissue partition coefficients. A few blood–tissue systems require a combination of two water–solvent partition coefficient measurements to model well (p < 0.01), namely: blood–muscle: chloroform and dibutyl ether, blood–liver: *N*-methyl-2-piperidone and ethanol/water (60:40) volume, and blood–kidney: DMSO and ethanol/water (20:80) volume.

**Conclusion:**

In vivo blood–tissue partition coefficients can be easily estimated through water–solvent partition coefficient measurements.

**Graphical abstract:**
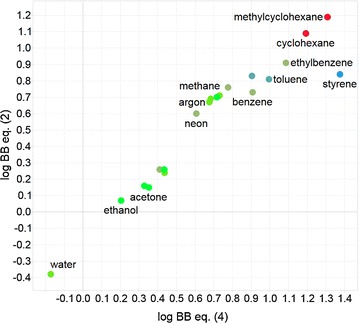
Predicted blood-brain barrier partition coefficients coloured by measured log BB value

**Electronic supplementary material:**

The online version of this article (doi:10.1186/s13065-015-0134-z) contains supplementary material, which is available to authorized users.

## Background

When a chemical enters the body, either through absorption or through direct administration, the relative concentrations found in the blood and other tissues are determined by physiochemical processes that separate the different parts of the body. For example, the blood–brain barrier separates the blood from the brain’s extracellular fluid in the central nervous system and protects the brain from potential neurotoxins and bacteria while allowing passage of essential molecules such as water, glucose, and amino acids that are crucial to neural function.

Knowing or predicting the partition coefficients (ratio of concentrations) of compounds between the bloodstream and various tissues is important in order to study the pharmacokinetic profile of drug candidates. While in vivo measurements are of most value, obtaining them is often not practical. Thus over the years several models have been developed to predict blood–tissue partition coefficients [1–3], with recent special attention being paid to the blood–brain barrier [4, 5].

Linear free energy relationships, developed by Abraham [6], have been applied directly to blood–tissue partition coefficients by Abraham, Gola, Ibrahim, Acree, and Liu [1] resulting in the model

1 $${\text{log BB}} = c + e E + s S + a A + b B + v V + i l_{\text{c}}$$

where log BB is the base ten logarithm of the blood–brain partition coefficient; E, S, A, B, and V are the standard solute descriptors [7, 8] and c, e, s, a, b, v, and i are the process coefficients, see Table 1. The descriptor I_c_ is an indicator variable for carboxylic acids that is taken to be one if the solute is a carboxylic acid and zero otherwise. This flag is not usually included in a general Abraham-type model but is needed here because the pH of blood is 7.4 and carboxylic acids are ionized at this pH.

**Table 1:** Coefficients in equation one for in vivo processes at 37 °C [1]

Process	c	e	s	a	b	v	i
Blood–brain	0.547	0.221	−0.604	−0.641	−0.681	0.635	−1.216
Blood–muscle	0.082	−0.059	0.010	−0.248	0.028	0.110	−1.022
Blood–liver	0.292	0.000	−0.296	−0.334	0.181	0.337	−0.597
Blood–lung	0.269	0.000	−0.523	−0.723	0.000	0.720	−0.988
Blood–kidney	0.494	−0.067	−0.426	−0.367	0.232	0.410	−0.481
Blood–heart	0.132	−0.039	−0.394	−0.376	0.009	0.527	−0.572
Blood–fat	0.077	0.249	−0.215	−0.902	−1.523	1.234	−1.013
Blood–skin	−0.105	−0.117	0.034	0.000	−0.681	0.756	−0.816
Water–skin	0.523	0.101	−0.076	−0.022	−1.951	1.652	0.000
Skin permeation	−5.420	−0.102	−0.457	−0.324	−2.608	2.066	0.000

Abraham and Acree have also used Eq. (1) to show that the water–1,9-decadiene system can be used as an excellent model for permeation through egg lecithin bilayers [9]. This suggests that other water–solvent systems could be used as models for blood–tissue coefficients. This would be very useful, because then in vivo blood–tissue partition coefficients could be estimated in vitro.

## Methods

Abraham model coefficients have been determined for over 90 organic solvents and can be predicted for others [10]. To find water–solvent systems that could be used to approximate blood–tissue systems we regressed the e, s, a, b, and v coefficients for each of the 90 organic solvents against the e, s, a, b, and v coefficients for each blood–tissue system listed in Table 1 above. The c-coefficient was not included as it is the intercept and could be adjusted separately after the regression had been performed. Specifically, we used linear regression in R (v 3.1.1)—‘lm’ command—and determined the best fit by using ‘regsubsets’ command in the ‘leaps’ package.

For example, the logarithm of partition coefficient for the blood–brain barrier is:

2 $$\begin{aligned} {\text{log BB}}& = 0.547 + 0.221 {\text{ E}} - 0.604 {\text{ S}} \\ &\quad- 0.641 {\text{ A}} - 0.681 {\text{B}} + 0.635 {\text{V}} - 1.216 {\text{ l}}_{\text{c}} \end{aligned}$$

Regressing Abraham solvent coefficients against this equation, we find that the water–methylcyclohexane partition system

3 $$\begin{aligned} {\text{log P}}_{\text{mcy}} &= 0.246 + 0.782 {\text{ E}} -1.982 {\text{S}} \\ &\quad- 3.517 {\text{ A}} - 4.293 {\text{B}} + 4.528 {\text{V}} \end{aligned}$$

can be used as a good (p < 0.002, R^2^ = 0.94) model for blood–brain barrier partition coefficients as follows:

4 $${\text{log BB}} = 0.505 + 0.169 {\text{log P}}_{\text{mcy}} - 1.216 {\text{ I}}_{\text{c}}$$

where log P_mcy_ is the measured log P value for methylcyclohexane. For additional details, datasets, and the R-code used, see the Open Notebook lab page [11].

Substituting Eq. (3) into (4) gives:

5 $$\begin{aligned} {\text{log BB}} &= 0.547 + 0.132 {\text{ E}} - 0.335 {\text{S}} \\ &\quad- 0.594 {\text{A}} - 0.726 {\text{ B}} + 0.765 {\text{ V}} - 1.216 {\text{ l}}_{\text{c}} \end{aligned}$$

**Fig. 1:**
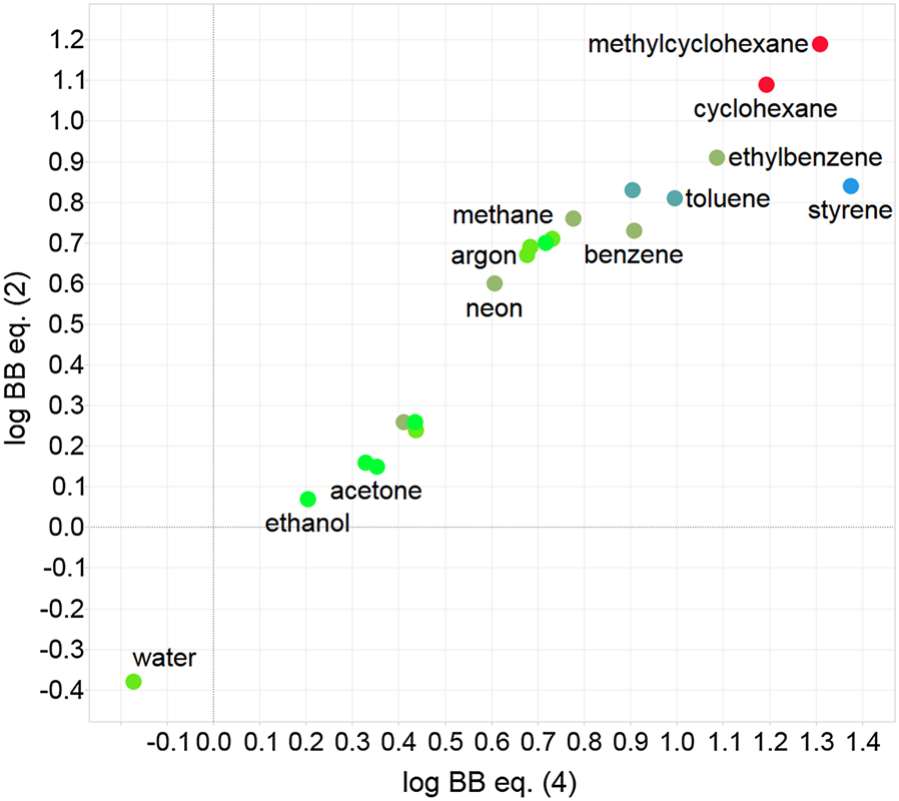
Predicted blood–brain barrier partition coefficients coloured by measured log BB value

Comparing Eqs. (2) and (5) we see fairly good agreement between coefficients. To validate our model we plotted the predicted log BB values for water, for six inorganic gases and for 13 common organic compounds using both equations, see Table 2; Additional file 1: Appendix Table S1; Fig. 1.

**Table 2:** Predicted blood–brain barrier partition coefficients

Compound	E	S	A	B	V	log BB Eq. (2)	log P_mcy_	log BB Eq. (4)
Water	0.00	0.45	0.82	0.35	0.167	−0.38	−4.01	−0.17
Ethanol	0.25	0.42	0.37	0.48	0.449	0.07	−1.78	0.20
1-Propanol	0.24	0.42	0.37	0.48	0.590	0.16	−1.05	0.33
Acetone	0.18	0.70	0.04	0.49	0.547	0.15	−0.90	0.35
t-Butanol	0.18	0.30	0.31	0.60	0.731	0.26	−0.56	0.41
2-Methyl-1-propanol	0.22	0.39	0.37	0.48	0.731	0.26	−0.41	0.44
1-Butanol	0.22	0.42	0.37	0.48	0.731	0.24	−0.40	0.44
Neon	0.00	0.00	0.00	0.00	0.085	0.60	0.60	0.61
Argon	0.00	0.00	0.00	0.00	0.190	0.67	1.02	0.68
Nitrogen	0.00	0.00	0.00	0.00	0.222	0.69	1.06	0.68
Krypton	0.00	0.00	0.00	0.00	0.246	0.70	1.25	0.72
Methane	0.00	0.00	0.00	0.00	0.250	0.71	1.34	0.73
Xenon	0.00	0.00	0.00	0.00	0.329	0.76	1.61	0.78
Sulphur Hexafluoride	–0.60	–0.20	0.00	0.00	0.464	0.83	2.36	0.90
Benzene	0.61	0.52	0.00	0.14	0.716	0.73	2.38	0.91
Toluene	0.60	0.52	0.00	0.14	0.857	0.81	2.90	1.00
Ethylbenzene	0.61	0.51	0.00	0.15	0.998	0.91	3.44	1.09
Cyclohexane	0.31	0.10	0.00	0.00	0.845	1.09	4.07	1.19
Methylcyclohexane	0.24	0.06	0.00	0.00	0.986	1.19	4.75	1.31
Styrene	0.85	0.65	0.00	0.16	0.955	0.84	5.15	1.38

The mean-square-error (MSE) between Eqs. (2) and (4) is 0.03 log units. The largest error occurs for styrene (AE 0.93 log units). In fact, without styrene, the MSE would drop to 0.02 log units. The reason why styrene is an outlier is that it is on the edge on the training-set chemical space. It has E and S values of 0.85 and 0.65 respectively as compared to the average values of E and S for the other compounds in the training set of 0.16 and 0.24 respectively. Other solvents that could be used as model systems for the blood–brain barrier include 1,9-decadience and octane.

We have modeled log BB indirectly by comparing the Abraham coefficients for water–solvent systems to the Abraham coefficients for log BB. We found that the water–methylcyclohexane system may be a good system to use to approximate log BB values in vitro, especially for solutes whose descriptor values fall within the range covered by both Abraham models (log BB and log P_mcy_). That is, Eq. (4) can be used to predict log BB values from log P_mcy_ values but should be used with caution when using it with compounds outside the chemical space used to create these models. In addition, the MSE of 0.03 is between Eqs. (2) and (4) and we do not claim that Eq. (4) will have this type of performance when used to predict measured log BB values. Our work indicates that methylcyclohexane is a good candidate for approximating log BB values but future work should focus on modeling log BB directly from log P_mcy_ when measured values for both log BB and log P_mcy_ are known for a significant number of compounds. Of particular interest would be experimentally determining both log BB and log P_mcy_ values for more common organic compounds (including crystalline compounds) that span a larger range of solute descriptors. The 20 compounds that are common to both the log BB and log P_mcy_ databases are inorganic gases and liquid organic compounds. The organic compounds, while not pharmaceutical compounds, are ones that workers are exposed to in chemical manufacturing processes.

## Results and discussion

We have seen that methylcyclohexane can be used to approximate log BB using Eq. (4). In general, we approximate the blood–tissue partition coefficient using the following equation

6 $${\text{log P}}_{\text{blood/tissue}} = {\text{c}}_{0} + {\text{c}}_{ 1} {\text{X}}_{ 1} + {\text{I}}_{\text{c}}$$

where c_0_ is the intercept, c_1_ is the coefficient multiplier for the log P system corresponding to solvent X_1_, and I_c_ is the carboxylic acid flag. Performing a similar analysis as described above and regressing the water–solvent system Abraham coefficients against the blood–tissue systems given in Table 1, we find the following results, presented in tables, see Tables 3, 4, 5, 6, 7, 8, 9, 10, 11, 12, where the p-values are the standard p-values from linear regression—calculated using the ‘lm’ command in R.

**Table 3:** Top five solvents for blood–brain

Solvent	c_0_	c_1_	p	R^2^
Methylcyclohexane	0.505	0.169	0.001	0.94
Octane	0.510	0.160	0.002	0.92
1,9-Decadiene	0.529	0.173	0.003	0.92
Cyclohexane	0.522	0.157	0.003	0.91
Decane	0.517	0.160	0.003	0.91

**Table 4:** Top five solvents for blood–muscle

Solvent	c_0_	c_1_	p	R^2^
Chloroform	0.077	0.028	0.2	0.41
2,2,2-Trifluoroethanol	0.063	0.047	0.2	0.38
Dichloromethane	0.074	0.025	0.2	0.35
Carbon tetrachloride	0.078	0.022	0.2	0.34
Iodobenzene	0.086	0.022	0.2	0.32

**Table 5:** Top five solvents for blood–liver

Solvent	c_0_	c_1_	p	R^2^
2,2,2-Trifluoroethanol	0.250	0.107	0.2	0.44
Methylcyclohexane	0.281	0.045	0.2	0.33
1,9-Decadiene	0.287	0.044	0.3	0.30
Chloroform	0.283	0.048	0.3	0.28
2,2,4-Trimethylpentane	0.279	0.040	0.3	0.27

**Table 6:** Top five solvents for blood–lung

Solvent	c_0_	c_1_	p	R^2^
2,2,2-Trifluoroethanol	0.165	0.263	0.04	0.69
Methylcyclohexane	0.239	0.122	0.06	0.64
1,9-Decadiene	0.256	0.124	0.07	0.60
Chloroform	0.243	0.135	0.08	0.58
2,2,4-Trimethylpentane	0.233	0.113	0.08	0.58

**Table 7:** Top five solvents for blood–kidney

Solvent	c_0_	c_1_	p	R^2^
2,2,2-Trifluoroethanol	0.443	0.129	0.2	0.40
Methylcyclohexane	0.481	0.053	0.3	0.29
1,9-Decadiene	0.489	0.052	0.3	0.26
Chloroform	0.483	0.055	0.3	0.23
2,2,4-Trimethylpentane	0.479	0.046	0.3	0.23

**Table 8:** Top five solvents for blood–heart

Solvent	c_0_	c_1_	p	R^2^
2,2,2-Trifluoroethanol	0.062	0.177	0.03	0.72
Methylcyclohexane	0.113	0.079	0.07	0.61
1,9-Decadiene	0.124	0.081	0.08	0.59
2,2,4-Trimethylpentane	0.109	0.073	0.09	0.54
Octane	0.115	0.072	0.09	0.54

**Table 9:** Top five solvents for blood–skin

Solvent	c_0_	c_1_	p	R^2^
Ethanol/water(10:90)vol	0.192	1.716	0.0002	0.98
N,N-Dimethylformamide	−0.058	0.153	0.0002	0.98
Ethanol/water(20:80)vol	0.099	0.811	0.0004	0.97
Ethanol/water(70:30)vol	−0.120	0.234	0.0005	0.96
Ethanol/water(30:70)vol	0.034	0.517	0.0006	0.96

**Table 10:** Top five solvents for blood–fat

Solvent	c_0_	c_1_	p	R^2^
Carbon disulfide	0.065	0.256	0.000001	0.998
Ethylbenzene	0.049	0.297	0.00002	0.99
*p*-Xylene	0.028	0.297	0.00002	0.99
*o*-Xylene	0.052	0.298	0.00002	0.99
Peanut oil	−0.128	0.358	0.0008	0.95

**Table 11:** Top five solvents for water–skin

Solvent	c_0_	c_1_	p	R^2^
THF	0.438	0.383	0.000003	0.997
Dibutylformamide	0.389	0.403	0.00002	0.99
1,4-Dioxane	0.474	0.401	0.00002	0.99
Acetone	0.395	0.410	0.00003	0.99
N-Formylmorphine	0.538	0.475	0.00003	0.99

**Table 12:** Top five solvents for skin-permeation

Solvent	c_0_	c_1_	p	R^2^
Methyl tert-butyl ether	−5.588	0.492	0.00002	0.99
THF	−5.532	0.501	0.0002	0.99
Diethyl ether	−5.596	0.503	0.0004	0.99
Ethanol/water(40:60)vol	−5.147	1.237	0.001	0.94
Ethanol/water(30:70)vol	−4.967	1.683	0.002	0.94

Examining the results presented in the Tables 3, 4, 5, 6, 7, 8, 9, 10, 11, 12, we see that the blood–brain barrier system can be modeled well with multiple solvents, including methylcyclohexane, octane, and 1,9-decadiene.

The results for blood–muscle and blood–liver were similar, with similar solvents, but very poor R^2^ values overall. The highest R^2^ was 0.44, exhibited by 2,2,2-trifluoroethanol for the blood–liver system.

The results for modeling the blood–lung, blood–kidney, and blood–heart partition coefficients were interesting as the top three suggested replacement solvents were identical, namely: 2,2,2-trifluoroethanol, methylcyclohexane, and 1,9-decdiene. The R^2^ values for these systems ranged between 0.41 for blood–kidney to 0.72 for blood–heart.

The blood–skin barrier model showed very strong results, with all of the top 5 R^2^ values above 0.95, which is very good. Some previously unseen solvents came up, the various ethanol–water mixtures composed four of the top five solvents.

Modeling the blood–fat system also had some very promising results. The highest was carbon disulfide with an R^2^ of 0.998. The lowest of the top 5 values was still very good, an R^2^ value of 0.95 for peanut oil. We suggest using the water/peanut oil system as a replacement system for blood–fat partition coefficients.

The water–skin solvents tested also produced strong results; the lowest of the top five R^2^ values is over 0.9, much higher than several of the earlier systems. Tetrahydrofuran resulted in the highest R^2^ value at 0.997.

The top five suggested replacement water–solvent systems for skin-permeation, like many previous blood–tissue systems, show great promise. The top three solvents being methyl tert-butyl ether, tetrahydrofuran, and diethyl ether.

Whilst most blood–tissue systems can be modeled with a single water–solvent system, blood–muscle, blood–liver, and blood–kidney had poor results, with R^2^ values all below 0.45. This is due to these three solvents having the smallest v values (0.110, 0.337, and 0.410) and highest b values (0.028, 0.181, 0.232) taking them out of the chemical space for single solvents. For these systems we modeled the blood–tissue coefficients using two measured water–solvent partition coefficient values X_1_ and X_2_ as follows

7 $${\text{log P}}_{\text{blood/tissue}} = {\text{c}}_{0} + {\text{c}}_{ 1} {\text{X}}_{1} + {\text{c}}_{ 2} {\text{X}}_{ 2} + {\text{I}}_{\text{c}}$$

where again c_0_ is the intercept. The results of these models are again presented in table form, see Tables 13, 14, 15.

**Table 13:** Top five results for two-variable blood–kidney partition coefficient

Solvent 1	Solvent 2	c_0_	c_1_	c_2_	p	R^2^
Ethanol/water(20:80)vol	DMSO	0.924	2.035	−0.428	0.0001	0.998
Ethanol/water(30:70)vol	DMSO	0.754	1.268	−0.417	0.001	0.99
Ethanol/water (40:60)vol	DMSO	0.617	0.916	−0.410	0.001	0.99
2-Butanol	Tributyl phosphate	0.408	0.799	−0.698	0.002	0.99
Ethanol/water(20:80)vol	Formamide	1.014	2.596	−0.786	0.03	0.90

**Table 14:** Top five results for two-variable blood–liver partition coefficient

Solvent 1	Solvent 2	c_0_	c_1_	c_2_	p	R^2^
Ethanol/water(60:40)vol	*N*-Methyl-2-piperidone	0.336	0.609	−0.352	0.002	0.99
Ethanol/water(80:20)vol	*N*-Methyl-2-piperidone	0.228	0.477	−0.327	0.005	0.97
Ethanol/water(90:10)vol	*N*-Methyl-2-piperidone	0.205	0.429	−0.315	0.008	0.96
Ethanol/water(70:30)vol	*N*-Ethylformamide	0.366	0.806	−0.566	0.01	0.94
Octadecanol	*N*-Methylpyrrolidinone	0.362	0.307	−0.278	0.02	0.92

**Table 15:** Top five results for two-variable blood–muscle partition coefficient

Solvent 1	Solvent 2	c_0_	c_1_	c_2_	p	R^2^
Chloroform	Dibutyl ether	0.075	0.113	−0.081	0.006	0.97
2,2,4-Trimethylpentane	1-Hexadecane	−0.011	0.453	−0.450	0.04	0.88
2,2,4-Trimethylpentane	Nonane	0.000	0.939	−0.912	0.04	0.88
1-Butanol	Ethylene glycol	−0.037	0.216	−0.310	0.1	0.75
1-Heptanol	Ethylene glycol	−0.002	0.185	−0.287	0.1	0.72

Blood–kidney regression with 1-variable produced very poor results, the top R^2^ value was 0.4 for 2,2,2-trifluoroethanol. Two variables can be used to increase the R^2^ value. This greatly improved all values for blood–kidney, the top value produced by a mixture of ethanol/water (20:80) and DMSO, with an R^2^ value of 0.997.

Blood–liver also produced very poor 1-variable results, so 2-variables were used to improve the R^2^ value. The highest R^2^ with 1-variable was 0.44 with 2,2,2-trifluoroethanol. The highest R^2^ with 2-variables was 0.99 by ethanol/water (60:40) and *N*-methyl-2-piperidone.

For the blood–muscle process, the overall 2-variable correlation coefficients were fairly good. The solvents that are best are chloroform and dibutyl ether with an R^2^ value of 0.97.

Combining two measured water/solvent partition coefficients can also improve the models for approximation the other blood–tissue partition coefficient values. See the Wiki page in the references for a complete list of all two-variable data tables [11].

When looking at the results, we note that the standard 1-octanol/water partition coefficient (log P) does not appear as a top solvent for any of the blood–tissue processes. This is interesting because log P has for a long time been assumed to be useful in estimating the distribution of drugs within the body and is a standard descriptor used in most QSAR modeling. Since the use of log P is prevalent throughout the chemistry community, we calculated how well the Abraham model for every blood–tissue partition coefficient can be modelled by the Abraham model for log P, see Table 16.

**Table 16:** Equation (6) coefficients for 1-octanol against multiple processes

Process	p	R^2^	c_0_	c_1_
Blood–brain	0.06	0.64	0.553	0.171
Blood–muscle	0.7	0.03	0.082	0.008
Blood–liver	0.6	0.07	0.293	0.026
Blood–lung	0.3	0.27	0.272	0.097
Blood–kidney	0.6	0.07	0.495	0.032
Blood–heart	0.3	0.31	0.134	0.070
Blood–skin	0.003	0.92	−0.100	0.161
Blood–fat	0.01	0.82	0.088	0.324
Water–skin	0.0003	0.97	0.537	0.415
Skin-permeation	0.0004	0.97	−5.402	0.545

Examining Table 16, we see that log P can be used to approximate all blood–tissue partition coefficients and actually performs moderately well for estimating log BB, but poorly for blood–muscle and all other organs. However, log P seems like a reasonable measure for processes to do with chemicals entering into the body: blood–skin, blood–fat, water–skin, and skin-permeation. The latter observation is in accord with the published results of Cronin and coworkers [12, 13] who noted that the percutaneous adsorption of organic chemicals through skin is mediated by both the hydrophobicity (log P) and the molecular size of the penetrant.

The water/solvent systems that included methylcyclohexane and 1,9-decadiene were in the top 5 results for multiple regressions. In Tables 17 and 18 we present the Eq. (6) coefficients for methylcyclohexane and 1,9-decadience respectively. In some case the coefficients have low R^2^ values. Keeping that in mind, we have a two more ways (with better performance than log P for predicting the important log BB partition coefficient) that all blood–tissue partition coefficients can be approximated by a single water–solvent partition coefficient measurement.

**Table 17:** Equation (6) coefficients for methylcyclohexane against multiple processes

Process	p	R^2^	c_0_	c_1_
Blood–brain	0.001	0.94	0.505	0.169
Blood–muscle	0.2	0.32	0.077	0.021
Blood–liver	0.2	0.33	0.281	0.045
Blood–lung	0.06	0.64	0.239	0.122
Blood–kidney	0.3	0.29	0.481	0.053
Blood–heart	0.07	0.61	0.113	0.079
Blood–skin	0.05	0.65	−0.132	0.111
Blood–fat	0.0009	0.95	0.007	0.285
Water–skin	0.03	0.71	0.452	0.289
Skin-permeation	0.02	0.80	−5.519	0.403

**Table 18:** Equation (6) coefficients for 1,9-decadiene against multiple processes

Process	p	R^2^	c_0_	c_1_
Blood–brain	0.003	0.92	0.529	0.173
Blood–muscle	0.3	0.29	0.080	0.021
Blood–liver	0.3	0.30	0.287	0.044
Blood–lung	0.07	0.60	0.256	0.124
Blood–kidney	0.3	0.26	0.489	0.050
Blood–heart	0.08	0.59	0.124	0.080
Blood–skin	0.04	0.71	−0.117	0.120
Blood–fat	0.0005	0.96	0.046	0.297
Water–skin	0.02	0.76	0.491	0.311
Skin-permeation	0.01	0.84	−5.465	0.439

As we have seen, methylcyclohexane is a good solvent when used to model the blood–brain barrier process. For other processes, blood–fat and skin-permeation, it showed a reasonably good R^2^ value (over 0.80). However, blood–muscle, blood–liver, and blood–kidney showed really poor R^2^ values (all less than 0.33).

1,9-Decadiene was just as good of a solvent as methylcyclohexane for approximating multiple blood–tissue coefficients. Blood–brain, blood–fat, and skin-permeation all showed good R^2^ values over 0.80. Just as in the methylcyclohexane case, the processes blood–muscle, blood–liver, blood–kidney were not well modeled and 2-solvent models are needed for more accurate approximations.

The research presented in this paper was performed under standard Open Notebook Science conditions, where day-to-day results were posted online in as near to real time as possible. For addition details, the data files, and the R-code used to find model systems, see the Open Lab Notebook page [11].

## Conclusions

Replacement solvents for various blood–tissue processes are proposed based upon the Abraham general solvation linear free energy relationship (1). For example, the top five solvents for approximating the blood brain barrier partition coefficient are methylcyclohexane, 1,9-decadiene, octane, cyclohexane, and decane. The five best solvents for the other blood–tissue partition coefficients were also calculated and presented. For three systems: muscle, liver, and lung; two-solvent models were presented to improve accuracy. For 1-solvent models, two solvents regularly came up in the list of best solvents for many processes. The top two recurring solvents were methylcyclohexane and 1,9-decadiene. This suggests that a single water–solvent partition measurement could in either methylcyclohexane or 1,9-decadiene can be used to approximate several blood–tissue partition coefficients.

## Additional file

10.1186/s13065-015-0134-z Measured and predicted Log BB values for 20 organic compounds.

## Abbreviations

THF: tetrahydrofuran
DMSO: dimethyl sulfoxide
MSE: mean square error
BB: blood–brain
MCY: methylcyclohexane
